# Computational blueprint and stereochemical validation of a small peptide derived from *Lacticaseibacillus casei* VITCM05 targeting estrogen receptor alpha and human epidermal growth factor receptor 2

**DOI:** 10.3389/fbinf.2026.1918460

**Published:** 2026-08-20

**Authors:** G. R. Shree Kumari, Mohanasrinivasan Vaithilingam

**Affiliations:** School of Bio Sciences and Technology, Vellore Institute of Technology, Vellore, Tamil Nadu, India

**Keywords:** ADMET, antimicrobial peptide, breast cancer, computational biology, *Lacticaseibacillus casei*, molecular docking, molecular dynamics simulation, PEP-FOLD

## Abstract

**Background:**

Breast cancer remains one of the leading causes of cancer-related mortality among women worldwide, necessitating the development of safer and more targeted therapeutic strategies. This study computationally extends our previous experimental investigation of a peptide derived from *Lacticaseibacillus casei* by evaluating its interactions with two clinically relevant breast cancer targets, estrogen receptor alpha (ERα; PDB ID: 3ERT) and human epidermal growth factor receptor 2 (HER2; PDB ID: 1N8Z).

**Methods:**

The peptide structure was predicted using PEP-FOLD and its stereochemical quality was assessed using a Ramachandran plot. Molecular docking was performed against ERα and HER2, followed by molecular dynamics simulations to evaluate structural stability. Binding free energy, binding affinity, dissociation constant, principal component analysis (PCA), free energy landscape (FEL), molecular mechanics (MM)/Poisson–Boltzmann surface area (PBSA) calculations, and *in silico* ADMET and toxicity predictions were performed to comprehensively characterise peptide–protein interactions.

**Results:**

The predicted peptide model exhibited 84.8% of residues located in the most favoured regions, while 15.2% were located in additionally allowed regions of the Ramachandran plot, indicating satisfactory stereochemical quality. Molecular docking demonstrated favourable interactions with both ERα and HER2, with HER2 showing a marginally more favourable docking score. Molecular dynamics simulations indicated stable peptide–protein complexes throughout the simulation period, as supported by root mean square deviation (RMSD), root mean square fluctuation (RMSF), radius of gyration (Rg), solvent-accessible surface area (SASA), and hydrogen-bond analyses. MM/PBSA calculations predicted stronger binding for the HER2 (1N8Z) complex (ΔG = −28.83 kJ/mol) than for the ERα (3ERT) complex (ΔG = −11.65 kJ/mol), highlighting the complementary nature of docking and dynamic free-energy estimation, which produced different receptor rankings. PCA and FEL analyses further demonstrated stable conformational sampling for both complexes. ADMET predictions suggested favourable peptide-like physicochemical properties while identifying pharmacokinetic and toxicity parameters that require further experimental validation.

**Conclusion:**

This computational study suggests that the *L. casei*-derived peptide exhibits favourable predicted interactions with ERα and HER2 and forms structurally stable peptide–protein complexes under simulated physiological conditions. These findings provide a computational framework for prioritising this probiotic-derived peptide for subsequent experimental validation and further investigation as a potential peptide-based therapeutic candidate for breast cancer.

## 1 Background

Breast cancer is the most frequently diagnosed malignancy among women worldwide and remains one of the leading causes of cancer-related mortality. According to the Global Cancer Statistics 2022, approximately 2.3 million new breast cancer cases and 670,000 deaths were reported globally, with incidence continuing to increase across both developed and developing countries (Bray et al., 2024). Despite substantial advances in early diagnosis and targeted therapies, breast cancer continues to present a significant clinical challenge because conventional treatment modalities, including chemotherapy, radiotherapy, hormone therapy, targeted therapy, and surgery, are frequently associated with adverse effects such as systemic toxicity, development of multidrug resistance, cardiotoxicity, gastrointestinal complications, immunosuppression, and tumour recurrence (Harbeck et al., 2019; Waks and Winer, 2019). These limitations have encouraged the search for safer and more selective therapeutic alternatives.

Among emerging therapeutic candidates, bioactive peptides produced by probiotic lactic acid bacteria (LAB) have attracted considerable attention because of their diverse biological activities, including antimicrobial, immunomodulatory, antioxidant, and anticancer properties (Cotter et al., 2013; Daba and Elkhateeb, 2024). Several studies have reported that probiotic-derived peptides can inhibit cancer cell proliferation, induce apoptosis, and interfere with tumour-associated cellular processes while exhibiting comparatively low toxicity toward normal cells (Kaur and Kaur, 2015; Ghadiri et al., 2024). These characteristics have positioned microbial peptides as promising molecules for the development of peptide-based therapeutics (Hiago Bellaver et al., 2024; Niamah et al., 2024; Sung et al., 2021).

Among probiotic bacteria, *Lacticaseibacillus casei (Lacticaseibacillus casei)* has received considerable attention owing to its Generally Recognised as Safe (GRAS) status and its documented health-promoting properties (Hill et al., 2014). In our previous study, a peptide-producing *L. casei* strain (VITCM05) was isolated from fermented *Cuminum cyminum* and experimentally characterised. The peptide exhibited broad-spectrum antibacterial activity and favourable physicochemical characteristics, including its proteinaceous nature and thermostability (Siddique and Vaithilingam, 2022). Although these findings established the peptide as a biologically active molecule, its potential interactions with breast cancer-associated molecular targets have not been investigated.

This represents an important knowledge gap because the structural basis of the interaction between the *L. casei*-derived peptide and clinically relevant breast cancer proteins remains unknown. In particular, its predicted binding behaviour, conformational stability, energetic characteristics, and pharmacokinetic properties against major therapeutic targets have not been evaluated. Addressing these questions is essential for determining whether this experimentally identified peptide warrants further investigation as a potential peptide-based therapeutic candidate.

Human epidermal growth factor receptor 2 (HER2) and estrogen receptor alpha (ERα) were selected as molecular targets because of their well-established clinical importance in breast cancer. HER2 is overexpressed in approximately 15%–20% of invasive breast cancers and is associated with aggressive tumour behaviour, increased recurrence, and poor clinical outcome, making it an important target for precision oncology (Harbeck et al., 2019; Loibl et al., 2021). In contrast, ERα is expressed in nearly 70% of breast cancers and plays a central role in estrogen-dependent tumour growth, representing the principal target for endocrine therapy in hormone receptor-positive disease (Harbeck et al., 2019; Waks and Winer, 2019). Because these receptors represent two clinically important molecular subtypes of breast cancer, they provide suitable targets for investigating the predicted interactions of the *L. casei*-derived peptide.

The novelty of the present study lies in its computational extension of our previously published experimental work by providing a molecular-level evaluation of a peptide isolated from *L. casei* VITCM05 against clinically relevant breast cancer targets (Jannatul Firdous and Mohanasrinivasan, 2022). Unlike previous investigations that primarily focused on antimicrobial characterisation or the general health benefits of probiotic bacteria, the present study examines the predicted structural interactions of the peptide with HER2 and ERα using complementary computational approaches.

Based on this rationale, the present study investigates the predicted interactions of the *L. casei*-derived peptide with HER2 and ERα using an integrated structural bioinformatics workflow comprising peptide structure prediction, molecular docking, molecular dynamics simulations, molecular mechanics (MM)/Poisson–Boltzmann surface area (PBSA) binding free-energy analysis, principal component analysis (PCA), free energy landscape analysis, and *in silico* ADMET prediction. The findings provide structural and computational evidence to prioritise this peptide for future experimental validation and should be interpreted as predictive rather than as evidence of biological or therapeutic efficacy.

## 2 Materials and methods

### 2.1 Peptide selection and retrieval

The peptide investigated in the present study was selected from our previously published experimental work on *Lacticaseibacillus casei* VITCM05, in which it was identified by MALDI-TOF/MS analysis and partially characterised (Jannatul Firdous and Mohanasrinivasan, 2022). The peptide sequence used for all computational analyses comprised 37 amino acid residues: ELETKLPLP DRETRRIIED NFQKIQDEIN KIESQMKK. The peptide sequence was used as the input for subsequent computational analyses, including peptide structure prediction, molecular docking, molecular dynamics simulations, MM/PBSA binding free-energy analysis, principal component analysis, free energy landscape analysis, and ADMET prediction.

### 2.2 Selection of breast cancer molecular targets

Breast cancer-associated molecular targets were selected based on their clinical relevance, established roles in breast cancer progression, therapeutic importance, and the availability of experimentally resolved three-dimensional structures suitable for computational analyses. The biological significance and disease associations of candidate targets were evaluated through an extensive literature survey using PubMed, together with information retrieved from the UniProt, GeneCards, KEGG, and DisGeNET databases (Stelzer et al., 2016; Piñero et al., 2019; Bateman et al., 2023; Kanehisa et al., 2023). Based on these criteria (Table 1), six breast cancer-associated proteins were selected for initial virtual screening. Among them, HER2 and ERα were identified as the most clinically relevant therapeutic targets owing to their well-established roles in breast cancer biology and their importance across distinct molecular subtypes of the disease (Harbeck et al., 2019; Waks and Winer, 2019; Loibl et al., 2021).

**TABLE 1:** Selected target proteins towards Breast Cancer Panel (MCF-7, MDA-MB-231, and SKBR3) with PDB ID.

Target	PDB ID	Chain selection
Estrogen receptor α (ERα)	3ERT	A
Estrogen receptor α	1L2I	Chain A
HER2 receptor	3PP0	Chain A
HER2 extracellular domain	1N8Z	Chain C
EGFR kinase	3POZ	Chain A
PI3Kα	4JPS	Chain A, B

The experimentally determined three-dimensional structures of the selected proteins were retrieved from the Protein Data Bank (PDB) (Burley et al., 2021). The crystal structures corresponding to HER2 (PDB ID: 1N8Z) and ERα (PDB ID: 3ERT) were selected for detailed computational analyses based on their structural quality, resolution, and suitability for molecular docking and molecular dynamics simulations. Prior to docking, all protein structures were prepared by removing crystallographic water molecules, co-crystallized ligands, and other non-essential heteroatoms, followed by the addition of hydrogen atoms to obtain receptor structures suitable for computational analysis.

### 2.3 Homology modelling and structural validation

The three-dimensional structure of the 37-amino acid *L. casei*-derived peptide was predicted using the Peptide Folding (PEP-FOLD) 3.5 server, which employs a *de novo* modelling approach based on a structural alphabet, hidden Markov models, and coarse-grained force fields to generate peptide conformations (Lamiable et al., 2016). The top-ranked peptide model generated by PEP-FOLD was selected for subsequent computational analyses. The stereochemical quality of the predicted peptide structure was assessed using PROCHECK, and the backbone geometry was evaluated by Ramachandran plot analysis to examine the distribution of amino acid residues within the allowed conformational regions (Laskowski et al., 1993). The experimentally determined crystal structures of the selected breast cancer target proteins were retrieved from the Protein Data Bank (PDB) (Burley et al., 2021). Where crystal structures contained missing amino acid residues, the incomplete regions were reconstructed using the SWISS-MODEL server, employing the corresponding experimentally determined crystal structure as the template (Waterhouse et al., 2018). The reconstructed protein structures were subsequently renumbered according to the original PDB residue numbering using the ScientiFlow Renumbering Tool to ensure sequence consistency for molecular docking, molecular dynamics simulations, and subsequent structural analyses.

### 2.4 Virtual screening and protein–peptide docking

Virtual screening was performed against six breast cancer-associated protein targets to evaluate the binding potential of the 37-amino acid *L. casei*-derived peptide. The peptide sequence used for docking was ELETKLPLPDRETRRIIEDNFQKIQDEINKIESQMKK.

Protein–peptide docking was carried out using the HDOCK server, a hybrid docking platform that integrates template-based modelling with *ab initio* docking algorithms for predicting protein–peptide interactions (Yan et al., 2020). Prior to docking, receptor structures were prepared by removing crystallographic water molecules, co-crystallised ligands, and other non-essential heteroatoms, followed by the addition of hydrogen atoms. The predicted peptide structure obtained from PEP-FOLD was used as the ligand for all docking analyses. Docking calculations were performed against the six selected breast cancer-associated proteins, and the resulting complexes were ranked according to their docking scores. Based on the docking results, the complexes exhibiting the most favourable predicted binding interactions, namely, HER2 (PDB ID: 1N8Z) and ERα (PDB ID: 3ERT), were selected for subsequent molecular dynamics simulations and MM/PBSA binding free-energy analyses.

### 2.5 Molecular dynamics simulation

Based on the protein–peptide docking results, the complexes formed between the *L. casei*-derived peptide and HER2 (PDB ID: 1N8Z) and ERα (PDB ID: 3ERT) were selected for molecular dynamics (MD) simulations because they exhibited the most favourable docking scores and predicted binding interactions among the six screened breast cancer-associated protein targets (Maisuradze et al., 2009; Huang and MacKerell, 2013; Lemkul, 2018; Valdés-Tresanco et al., 2021). All molecular dynamics simulations were performed using GROMACS with the CHARMM36 force field. The protein–peptide complexes (3ERT + peptide and 1N8Z + peptide) were selected, and the protein–protein MD simulation with analysis pipeline mode was executed for the analysis of the selected apoproteins (3ERT and 1N8Z). Energy minimisation was performed using the steepest descent algorithm to eliminate steric clashes and unfavourable contacts. Production molecular dynamics simulations were carried out for 300 ns under periodic boundary conditions while maintaining constant temperature and pressure. Following completion of the simulations, the generated trajectories were used for subsequent analyses, including root mean square deviation (RMSD), root mean square fluctuation (RMSF), radius of gyration (Rg), solvent-accessible surface area (SASA), hydrogen bond analysis, PCA, free energy landscape (FEL) analysis, and MM/PBSA binding free-energy and DSSP calculations to evaluate the dynamic stability and interaction behaviour of the protein–peptide complexes (ScientiFlow, 2025).

### 2.6 Molecular mechanics Poisson–Boltzmann surface area analysis

The binding free energies of the protein–peptide complexes were estimated using the MM/PBSA method based on the equilibrated trajectories obtained from the 100 ns molecular dynamics simulations. MM/PBSA calculations were performed using snapshots extracted from the production trajectories to estimate the relative binding free energies of the HER2 (1N8Z)–peptide and ERα (3ERT)–peptide complexes. The total binding free energy (ΔG_binding) was calculated as the sum of the molecular mechanics energy contributions (van der Waals and electrostatic interactions) and solvation free-energy terms (polar and non-polar contributions). The resulting energy decomposition enabled evaluation of the major energetic components contributing to protein–peptide binding.

### 2.7 *In silico* ADMET and toxicity prediction

The pharmacokinetic and toxicity profiles of the *L. casei*-derived peptide were predicted using the ADMETlab 3.0 web server, an integrated computational platform for evaluating the absorption, distribution, metabolism, excretion, and toxicity (ADMET) properties of bioactive molecules (Dong et al., 2018). The peptide sequence was submitted to the server, and comprehensive predictions were obtained for its physicochemical characteristics, absorption, distribution, metabolic behaviour, excretion, medicinal chemistry properties, and toxicity profile. The predicted physicochemical properties included molecular weight, hydrogen bond donors and acceptors, topological polar surface area, lipophilicity, and molecular flexibility. Pharmacokinetic parameters comprised intestinal absorption, Caco-2 permeability, blood–brain barrier permeability, plasma protein binding, P-glycoprotein interaction, cytochrome P450 enzyme interaction, clearance, and elimination characteristics. Toxicological endpoints, including hepatotoxicity, nephrotoxicity, carcinogenicity, mutagenicity, cardiotoxicity, skin sensitisation, respiratory toxicity, eye irritation, and acute oral toxicity, were also evaluated. The generated predictions were subsequently used to assess the overall drug-likeness and safety profile of the peptide for comparative interpretation with the structural and energetic analyses.

## 3 Results

### 3.1 Peptide structure prediction and structural validation

**FIGURE 1:**
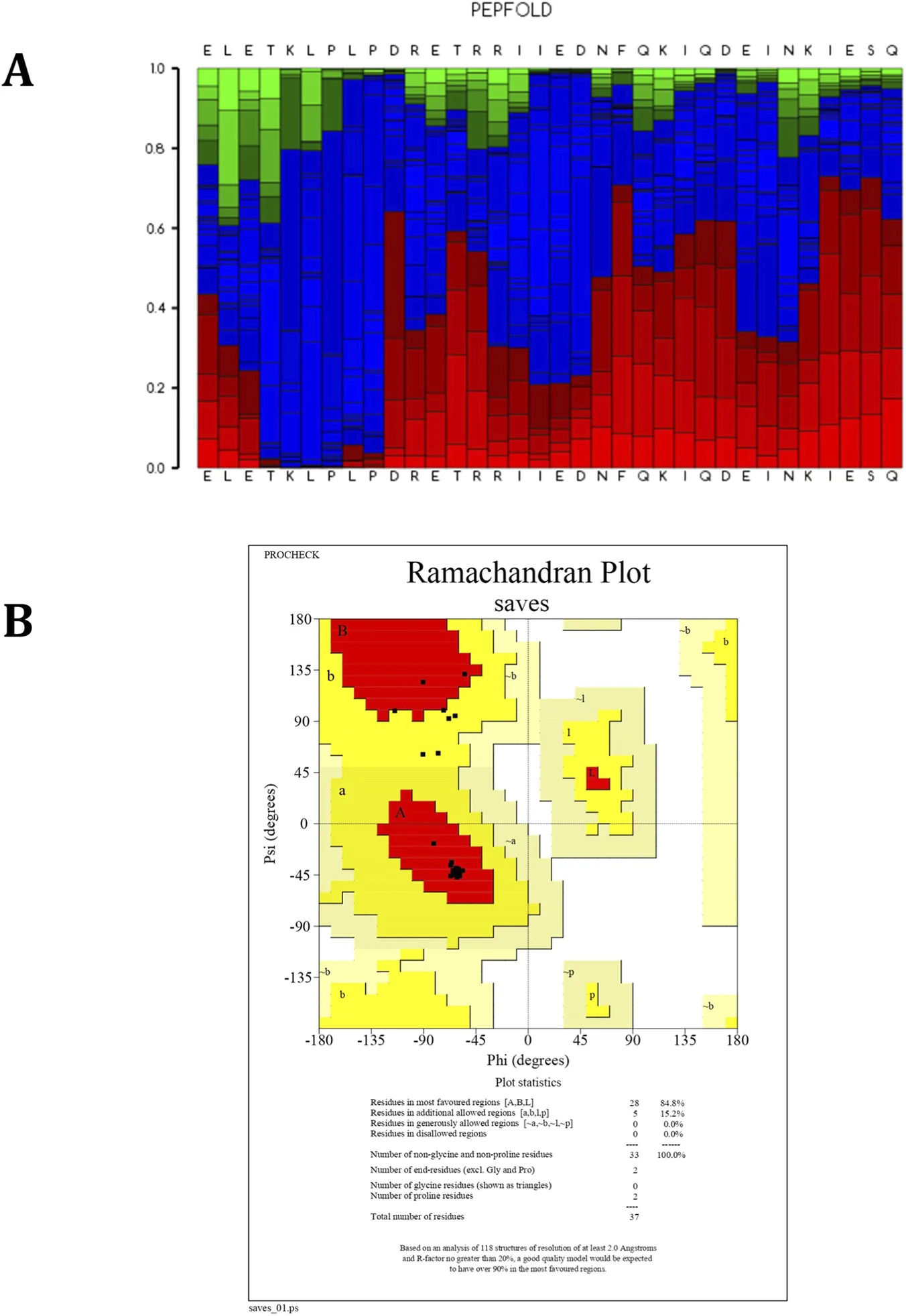
Structural prediction and stereochemical validation of the *Lacticaseibacillus casei* peptide. **(A)** Secondary structure prediction generated using PEP-FOLD showing the residue-wise structural propensity of the peptide. **(B)** Ramachandran plot generated using PROCHECK demonstrating the stereochemical quality of the predicted peptide model. A total of 84.8% of residues were located in the most favoured regions, while the remaining residues occupied additionally allowed regions, with no residues present in disallowed regions, indicating an acceptable structural model for subsequent computational analyses.

The three-dimensional structure of the 37-amino acid *L. casei*-derived peptide was predicted using the PEP-FOLD 3.5 server. Among the generated models, the top-ranked peptide model with the lowest sOPEP energy was selected for subsequent analyses (Table 2). The predicted structure exhibited a compact three-dimensional conformation with defined secondary structural elements (Figure 1A). The stereochemical quality of the selected peptide model was assessed using PROCHECK through Ramachandran plot analysis. A total of 84.8% of the residues were located within the most favoured regions, while the remaining 15.2% were distributed within the additionally allowed regions. No residues were observed in the generously allowed or disallowed regions, indicating acceptable stereochemical quality of the predicted peptide model (Figure 1B). The validated peptide structure was subsequently used for molecular docking and molecular dynamics simulations.

**TABLE 2:** Cluster statistics and energy distribution of the top-ranked peptide models predicted by PEP-FOLD.

PDB	Number in cluster	Center	Lowest energy
3ERT	124	−763.4	−736.6
1N8Z	116	−693.3	−737.6
1L2I	177	−660.9	−660.9
4JPS	171	−646.1	−722.3
3PPO	238	−588.3	−727.4
3POZ	129	−583.3	−627.6

### 3.2 Molecular docking, binding free energy, binding affinity, and dissociation constant prediction

**FIGURE 2:**
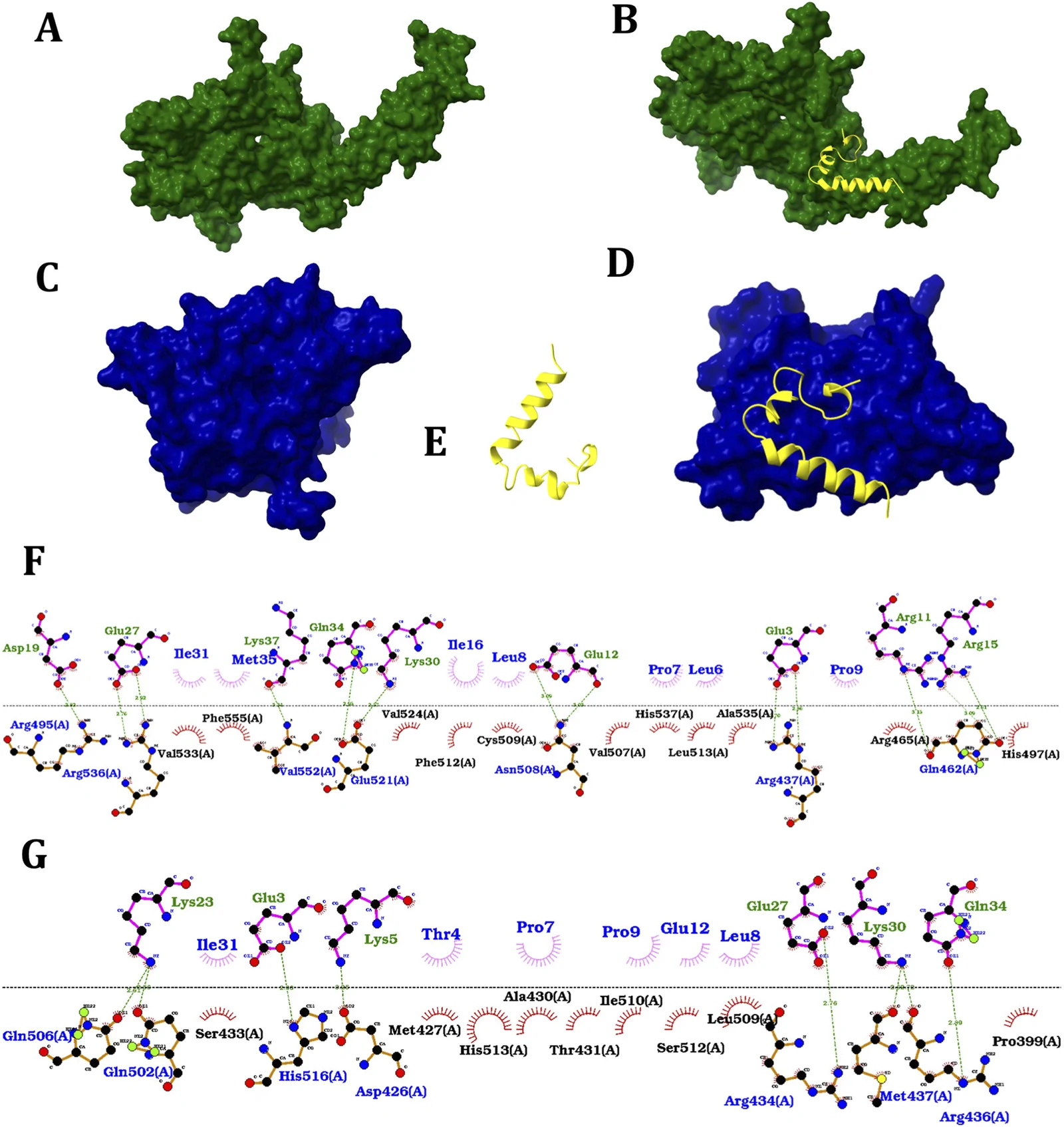
Predicted binding modes of the L. casei peptide with HER2 (1N8Z) and ERα (3ERT). **(A)** Surface representation of HER2 (1N8Z). **(B)** Docked complex of the peptide bound to HER2. **(C)** Surface representation of ERα (3ERT). **(D)** Docked complex of the peptide bound to ERα. **(E)** Three-dimensional structure of the L. casei peptide. **(F)** Two-dimensional interaction map of the peptide–HER2 complex highlighting hydrogen bonds and hydrophobic contacts. **(G)** Two-dimensional interaction map of the peptide–ERα complex illustrating key interacting residues involved in peptide recognition.

The protein–peptide docking analysis was performed against six breast cancer-associated molecular targets to evaluate the binding potential of the *L. casei*-derived peptide. The docking results demonstrated differential binding affinities among the screened proteins (Table 2). Based on the docking scores, the peptide exhibited the most favourable predicted interactions with ERα (PDB ID: 3ERT) and HER2 (PDB ID: 1N8Z). These two complexes were therefore selected for subsequent molecular dynamics simulations and MM/PBSA binding free-energy analyses. The docked complexes revealed that the peptide occupied the predicted binding pockets of both receptors through multiple intermolecular interactions, including hydrogen bonds and hydrophobic contacts (Figures 2A–G). Visual inspection of the docking poses indicated favourable geometric complementarity between the peptide and the receptor binding sites.

To further characterise the predicted protein–peptide interactions, the binding free energy (ΔG), binding affinity, and dissociation constant (Kd) were estimated from the docking results (Table 3). The ERα–peptide complex (3ERT) exhibited a predicted binding free energy of −8.76 kcal/mol, corresponding to a binding affinity of 6.67 × 10^6^ M^-1^ and a dissociation constant of 1.50 × 10^−7^ M. In comparison, the HER2–peptide complex (1N8Z) showed a predicted binding free energy of −8.60 kcal/mol, with a binding affinity of 5.10 × 10^6^ M^-1^ and a dissociation constant of 1.96 × 10^−7^ M. The remaining screened targets exhibited comparatively lower predicted binding affinities and were therefore not considered for further dynamic simulations. Overall, the molecular docking results identified ERα (3ERT) and HER2 (1N8Z) as the two highest-ranked protein–peptide complexes among the six screened breast cancer-associated targets, providing the basis for subsequent molecular dynamics and energetic analyses.

**TABLE 3:** MM/PBSA-derived binding free energy components for the peptide complexes with breast cancer-associated targets.

Energy component (kJ/mol)	1N8Z (mean ± SEM)	3ERT (mean ± SEM)
ΔVDWAALS	−51.59 ± 1.25	−19.25 ± 0.54
ΔEEL	−9.48 ± 1.64	−28.37 ± 2.01
ΔEGB	40.12 ± 2.18	38.91 ± 2.26
ΔESURF	−7.87 ± 0.19	−2.94 ± 0.08
ΔGGAS	−61.07 ± 2.66	−47.62 ± 2.51
ΔGSOLV	32.24 ± 2.01	35.97 ± 2.18
Δgtotal	−28.83 ± 0.72	−11.65 ± 0.35

### 3.3 Molecular dynamics simulation

#### 3.3.1 Root mean square deviation

**FIGURE 3:**
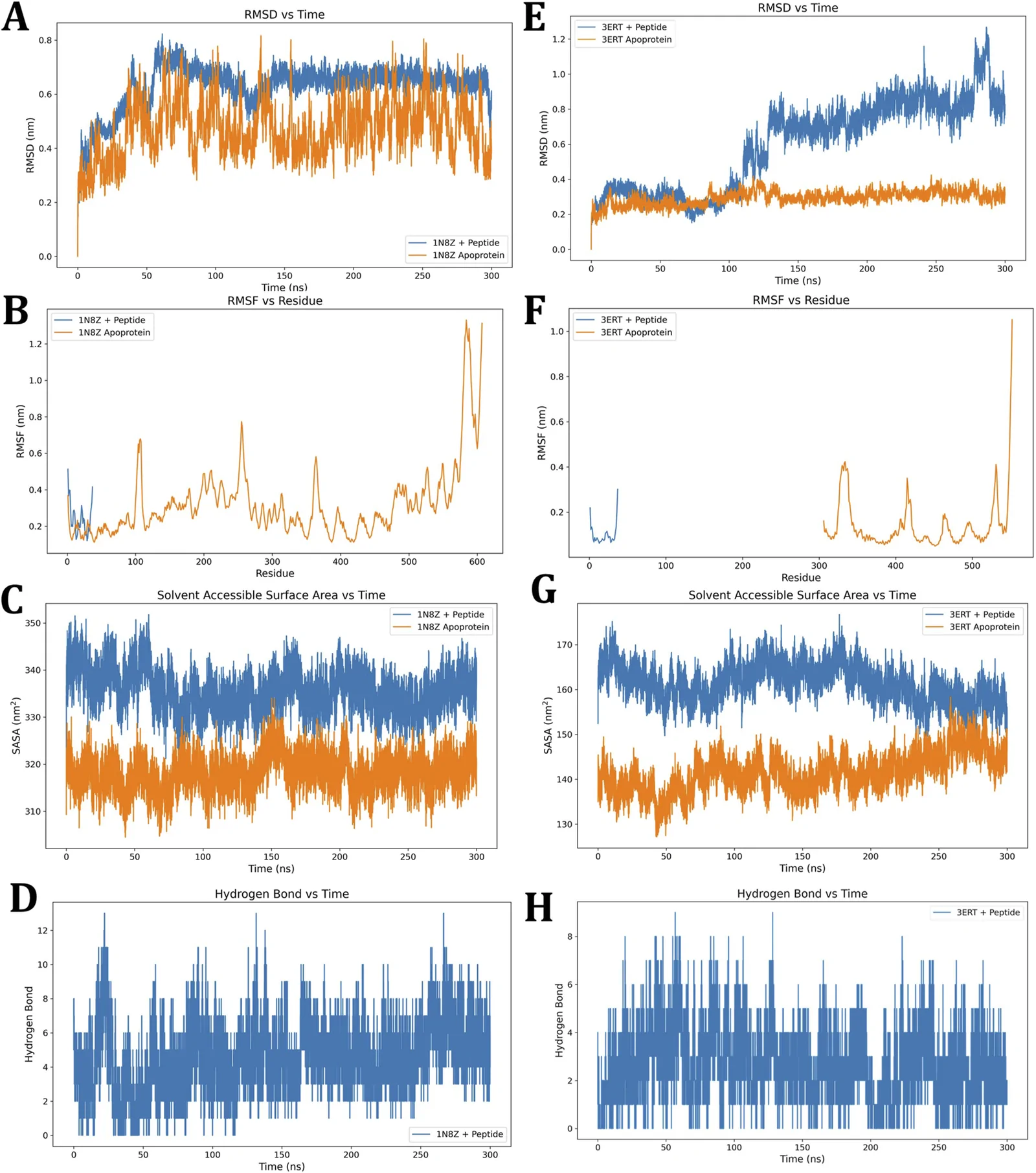
Molecular dynamics analysis of HER2 and ERα systems during 300 ns simulations. **(A)** RMSD of HER2 apo protein and HER2–peptide complex. **(B)** RMSF profiles of HER2 systems. **(C)** SASA of HER2 apo and peptide-bound systems. **(D)** Number of intermolecular hydrogen bonds formed between HER2 and the peptide throughout the simulation. **(E)** RMSD of ERα apo protein and ERα–peptide complex. **(F)** RMSF profiles of ERα systems. **(G)** SASA of ERα apo and peptide-bound systems. **(H)** Intermolecular hydrogen bonds observed between ERα and the peptide over the simulation period.

The structural stability of the peptide-bound HER2 (1N8Z) and ERα (3ERT) complexes during the 300 ns molecular dynamics simulations was evaluated using root mean square deviation (RMSD) analysis. Both complexes exhibited an initial increase in RMSD during the equilibration phase, followed by stabilisation throughout the remainder of the simulation (Figures 3A,E). The 1N8Z–peptide complex reached equilibrium after approximately 15 ns and fluctuated within a narrow RMSD range, whereas the 3ERT–peptide complex stabilised after approximately 20 ns with slightly higher fluctuations. The absence of large structural deviations following equilibration indicates that both complexes maintained stable conformations throughout the simulation period.

#### 3.3.2 Root mean square fluctuation

Residue-wise flexibility of the peptide-bound receptor complexes was assessed using RMSF analysis (Figures 3B,F). In both complexes, relatively low fluctuations were observed across most residues, whereas higher fluctuations were primarily confined to terminal regions and exposed loop segments. The core structural regions exhibited comparatively lower atomic mobility throughout the simulation. The overall RMSF profiles of the two complexes were comparable, with no pronounced fluctuations observed within the peptide-binding regions.

#### 3.3.3 Radius of gyration

**FIGURE 4:**
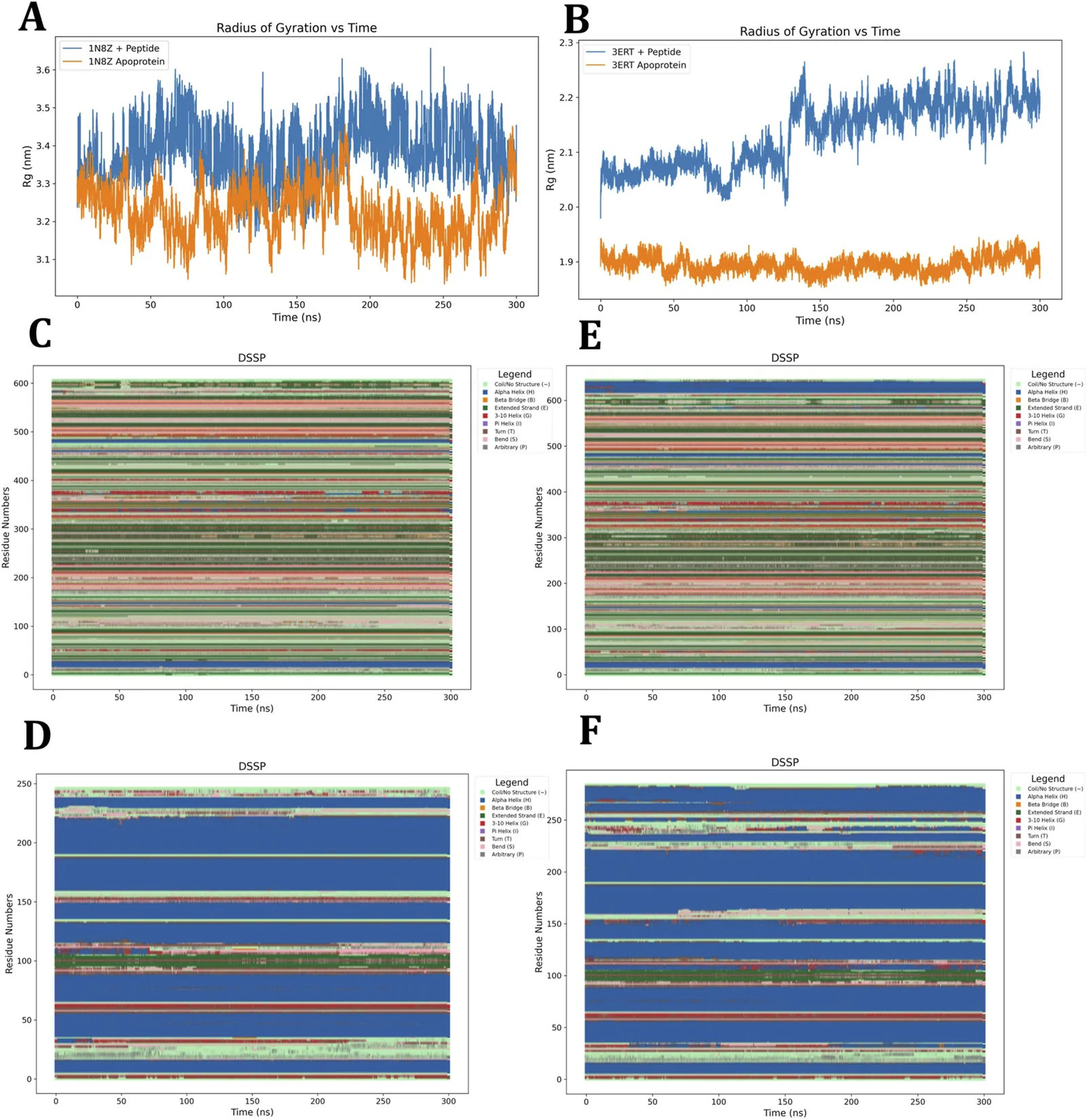
Structural compactness and secondary structure evolution during molecular dynamics simulations. **(A)** Radius of gyration (Rg) of HER2 apo and HER2–peptide systems. **(B)** Radius of gyration of ERα apo and ERα–peptide systems. **(C)** DSSP secondary structure analysis of HER2 apo protein. **(D)** DSSP analysis of HER2–peptide complex. **(E)** DSSP analysis of ERα apo protein. **(F)** DSSP analysis of ERα–peptide complex. The persistence of secondary structural elements indicates overall structural stability throughout the simulations.

The compactness of the protein–peptide complexes was evaluated using the Rg throughout the simulation (Figures 4A,B). Both complexes maintained relatively constant Rg values during the 300 ns trajectory, indicating preservation of their overall structural compactness. Minor fluctuations were observed during the equilibration phase; however, no substantial changes in molecular compactness occurred thereafter. These observations suggest that both complexes retained their folded conformations throughout the simulation.

#### 3.3.4 Solvent accessible surface area

The SASA of both protein–peptide complexes was monitored to evaluate changes in solvent exposure during the simulation (Figures 3C,G). The SASA profiles exhibited only moderate fluctuations over time, with both complexes maintaining relatively stable solvent-accessible surface areas throughout the trajectory. No abrupt increases or decreases in solvent exposure were observed, indicating the absence of major conformational rearrangements during the simulation.

#### 3.3.5 Hydrogen bond analysis

Hydrogen-bond interactions between the peptide and the receptor proteins were analysed throughout the simulation to assess the persistence of intermolecular interactions (Figures 3D,H). Both complexes maintained multiple hydrogen bonds during the 300 ns trajectory, although the number of hydrogen bonds fluctuated dynamically over time. These transient variations are consistent with the inherent flexibility of protein–peptide interactions during molecular dynamics simulations. Overall, continuous hydrogen-bond formation was observed throughout the trajectory, indicating sustained intermolecular contacts between the peptide and both receptors.

#### 3.3.6 Secondary structure analysis

The secondary structural stability of the peptide-bound HER2 (1N8Z) and ERα (3ERT) complexes was evaluated using the Dictionary of Secondary Structure of Proteins (DSSP) algorithm (Figures 4C–F). Throughout the 300 ns simulation, the predominant α-helical and β-sheet structural elements of both receptors remained largely conserved. Minor transitions between coil, turn, and bend conformations were observed mainly within flexible loop regions, whereas the overall secondary structure architecture remained unchanged. No extensive secondary structure loss or large-scale conformational rearrangements were detected during the simulation, indicating preservation of the native secondary structure organisation of both receptor complexes.

### 3.4 Principal component analysis

**FIGURE 5:**
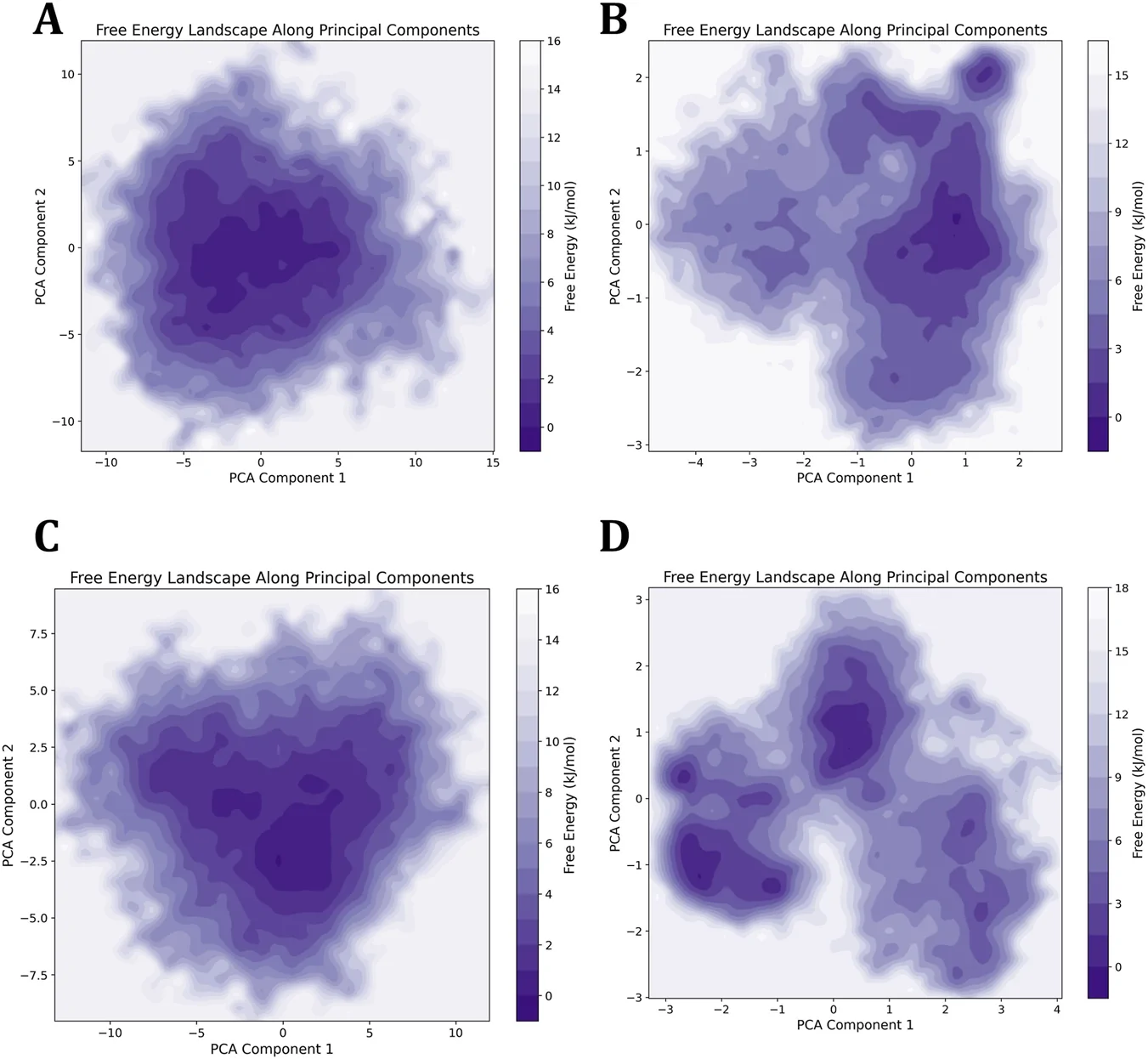
Two-dimensional free energy landscapes derived from principal component analysis. **(A)** HER2 apo protein. **(B)** HER2–peptide complex. **(C)** ERα apo protein. **(D)** ERα–peptide complex. Darker regions correspond to lower free-energy conformational basins representing energetically favourable states sampled during the molecular dynamics simulations.

PCA was performed to characterise the dominant collective motions and conformational sampling of the 3ERT systems during the 100 ns molecular dynamics simulations. The eigenvalue spectrum of the apo 3ERT system showed that the first three principal components contributed eigenvalues of 3.73028, 1.09459, and 0.944537 nm^2^, respectively, with a progressive decline in the contribution of the higher-order eigenvectors. The dominant contribution of PC1 indicates that the largest-amplitude collective motion of the receptor was primarily captured by the first principal mode, whereas the subsequent modes contributed progressively smaller components of the overall conformational variance. The rapid decrease in eigenvalues across the leading modes indicates that the major collective motions of the system were concentrated within a limited number of principal components (Figure 5).

The conformational ensembles sampled by the apo and peptide-bound 3ERT systems were further characterised by projection-based clustering. The apo-3ERT trajectory was predominantly represented by Cluster 1 (87.40%), whereas Cluster 2 accounted for 7.79% of the sampled conformations (Figure 10A). This distribution indicates that the apo receptor predominantly occupied a major conformational ensemble throughout the simulation, with limited sampling of an alternative structural state. The corresponding representative structures of the major apo conformational clusters further illustrated the structural states sampled by the receptor during the simulation (Figures 10C–F).

In contrast, the 3ERT–peptide complex exhibited a more distributed conformational ensemble. Cluster 1 accounted for 20.45% of the sampled conformations, followed by Cluster 2 (20.29%), Cluster 3 (9.33%), and Cluster 4 (7.69%) (Figure 10B). The broader distribution of cluster populations indicates that peptide association was accompanied by the sampling of multiple conformational states rather than a single predominant structural ensemble. The corresponding representative structures further illustrated the conformational states sampled by the peptide-bound receptor during the simulation (Figures 10G–J). Collectively, the PCA and clustering analyses indicated distinct conformational sampling behaviours between the apo and peptide-bound 3ERT systems (Figures 9, 10). The apo receptor was dominated by a major conformational ensemble, whereas the peptide-bound complex exhibited a more heterogeneous distribution of sampled structural states. These results indicate that peptide association altered the collective conformational dynamics and increased the diversity of conformational states accessed by the 3ERT receptor during the simulation.

### 3.5 Free energy landscape analysis

FEL analysis was performed to characterise the energetic distribution of the conformational states sampled by the apo and peptide-bound 3ERT systems. The resulting two-dimensional free-energy landscapes revealed the relative organisation of conformational states according to their associated free-energy values and identified the predominant low-energy regions sampled during the molecular dynamics simulations.

**FIGURE 6:**
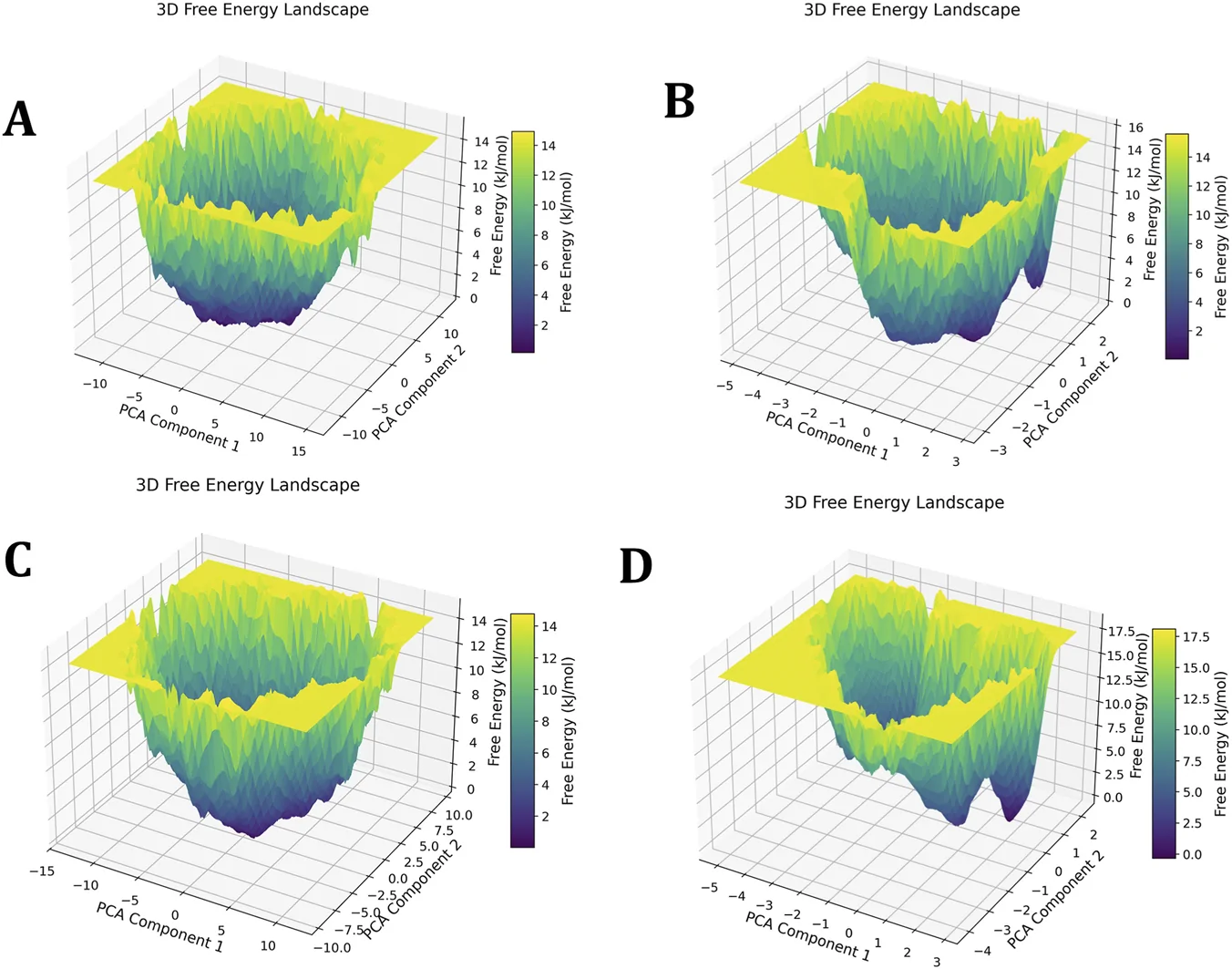
Three-dimensional free energy landscape of apo proteins and peptide-bound complexes. **(A)** HER2 apo protein. **(B)** HER2–peptide complex. **(C)** ERα apo protein. **(D)** ERα–peptide complex. The energy landscapes illustrate the conformational space explored during simulation and identify low-energy minima corresponding to stable structural states.

The apo 3ERT system exhibited a well-defined dominant low-free-energy region, indicating preferential occupation of a major energetically favourable conformational state during the simulation (Figure 6C). The organisation of the landscape was consistent with the predominance of Cluster 1 observed in the clustering analysis. The associated conformational representations illustrated the structural characteristics of the states sampled within the corresponding low-energy region (Figures 10D–F). In contrast, the 3ERT–peptide complex exhibited a more distributed free-energy landscape with multiple energetically favourable regions (Figure 10G). The presence of several low-energy regions indicates that the peptide-bound receptor accessed multiple energetically favourable conformational states during the simulation. The corresponding conformational representations of the peptide-bound system are shown in Figures 10H–J.

Comparison of the two systems demonstrated a distinct reorganisation of the conformational free-energy landscape following peptide association. On the other hand, the apo receptor was characterised by a predominant low-energy conformational region; the peptide-bound complex displayed multiple favourable regions within the sampled landscape. This distribution indicates that peptide binding modified the energetic accessibility of receptor conformations and promoted sampling of a broader set of energetically favourable states. Nevertheless, both systems remained within defined low-energy regions throughout the simulation, indicating that the sampled conformational ensembles were energetically favourable and structurally stable over the simulated timescale.

### 3.6 MM/PBSA binding free-energy analysis

**FIGURE 7:**
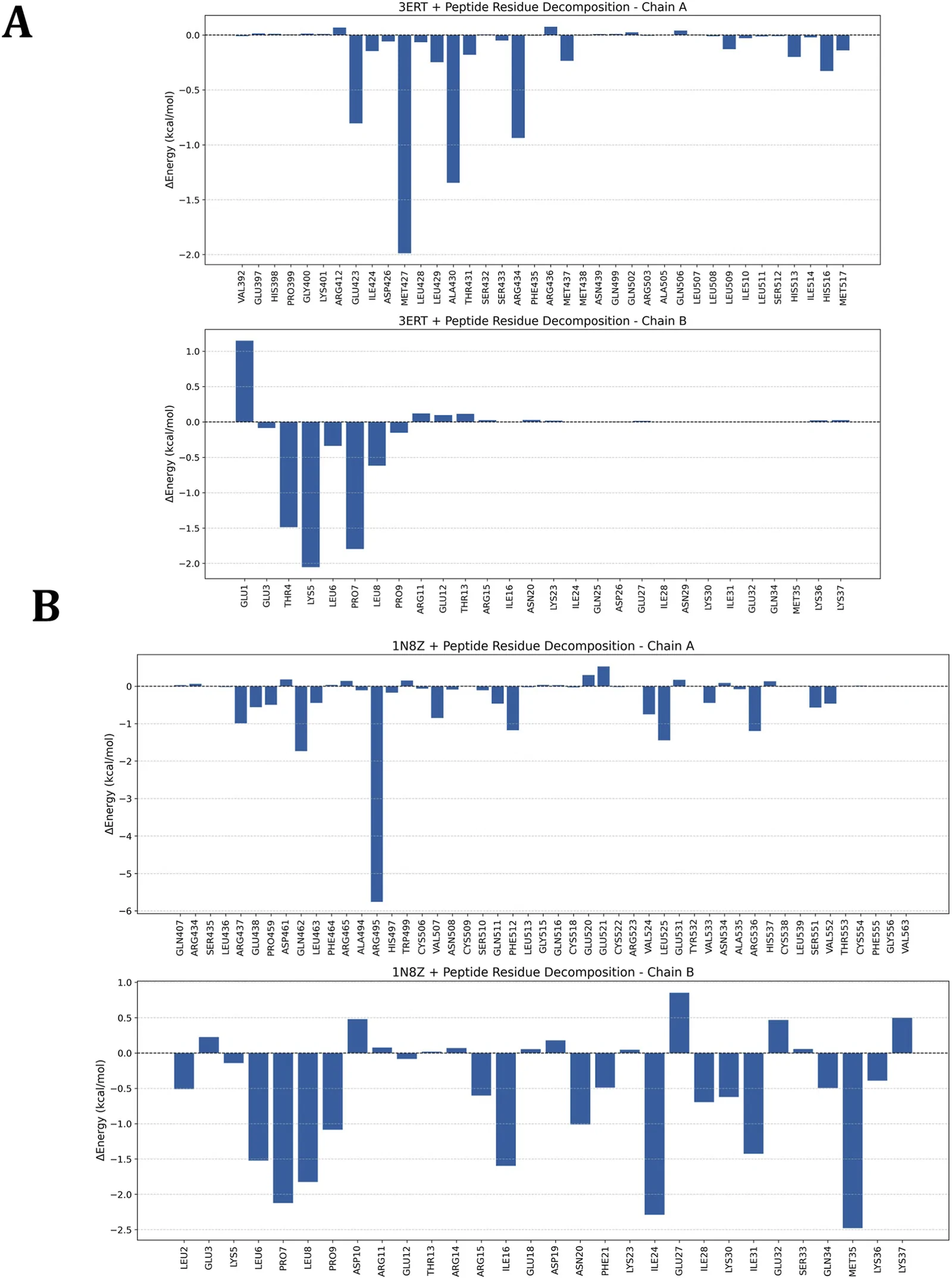
Per-residue MM/PBSA free-energy decomposition of peptide binding. **(A)** Residue-wise energy contribution for the ERα–peptide complex showing individual residues from receptor and peptide contributing to complex stabilization. **(B)** Residue-wise energy contribution for the HER2–peptide complex identifying favourable amino acid residues responsible for binding affinity. Negative values indicate energetically favourable interactions.

MM/PBSA binding free-energy calculations were performed using equilibrated snapshots extracted from the production phase of the 100 ns molecular dynamics trajectories for both protein–peptide complexes. The total binding free energies, together with their individual energetic components, including van der Waals, electrostatic, polar solvation, non-polar solvation, gas-phase, and solvation energies, are summarised in Table 3. Both complexes exhibited negative total binding free-energy (ΔG_TOTAL) values, indicating thermodynamically favourable peptide binding. However, the HER2 (1N8Z)–peptide complex displayed a more favourable overall binding free energy (ΔG_TOTAL = −28.83 ± 0.72 kJ/mol) than the ERα (3ERT)–peptide complex (ΔG_TOTAL = −11.65 ± 0.35 kJ/mol), suggesting stronger predicted binding following dynamic equilibration. To further identify the amino acid residues contributing to peptide recognition and complex stabilisation, per-residue MM/PBSA free-energy decomposition analysis was performed (Figures 7A,B). Several receptor residues exhibited favourable energy contributions to peptide binding in both complexes, indicating that peptide recognition was mediated through multiple stabilising residue-level interactions rather than a limited number of dominant contacts.

**FIGURE 8:**
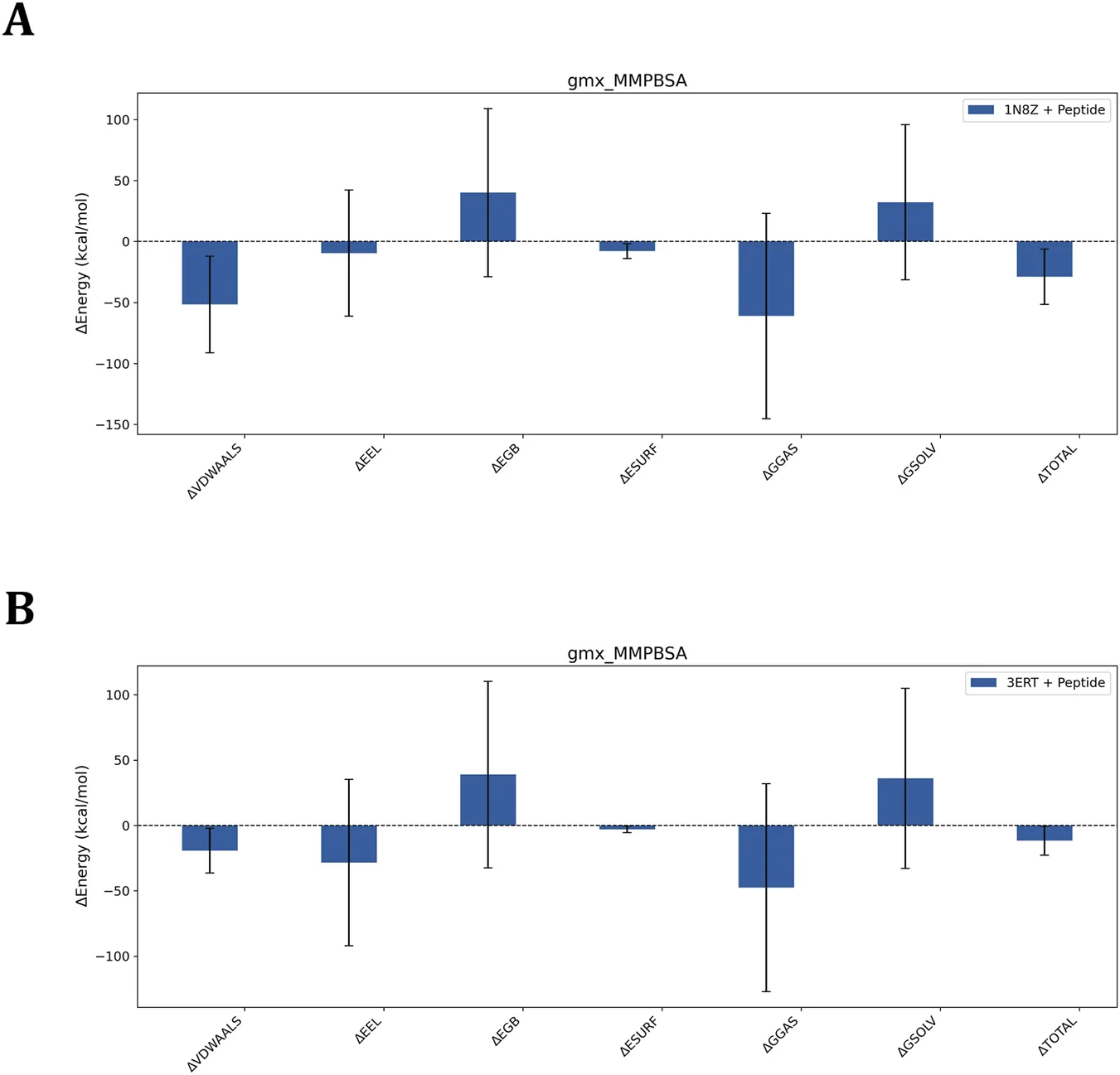
MM/PBSA binding free-energy component analysis for peptide–receptor complexes. **(A)** Energy decomposition of the HER2–peptide complex showing contributions from van der Waals interactions (ΔVDWAALS), electrostatic energy (ΔEEL), polar solvation (ΔEGB), non-polar solvation (ΔESURF), gas-phase energy (ΔGGAS), solvation energy (ΔGSOLV), and total binding free energy (ΔTOTAL). **(B)** Corresponding MM/PBSA energy components for the ERα–peptide complex. Error bars represent the standard deviation calculated from trajectory frames extracted during the equilibrated phase of the 100 ns molecular dynamics simulation.

The individual MM/PBSA energy components were further compared between the HER2 (1N8Z)–peptide and ERα (3ERT)–peptide complexes (Figures 8A,B). For the HER2 complex, binding was predominantly driven by favourable van der Waals interactions (ΔVDWAALS = −51.59 ± 1.25 kJ/mol), whereas electrostatic interactions made a comparatively smaller contribution (ΔEEL = −9.48 ± 1.64 kJ/mol). In contrast, the ERα complex exhibited a larger favourable electrostatic contribution (ΔEEL = −28.37 ± 2.01 kJ/mol) despite comparatively weaker van der Waals interactions (ΔVDWAALS = −19.25 ± 0.54 kJ/mol). In both complexes, favourable gas-phase interactions were partially offset by unfavourable polar solvation energies, whereas non-polar solvation contributed favourably to binding. Overall, the MM/PBSA analysis indicates that both receptor–peptide complexes are energetically favourable, with the HER2 complex exhibiting stronger predicted binding following molecular dynamics equilibration due primarily to more favourable van der Waals interactions.

**FIGURE 9:**
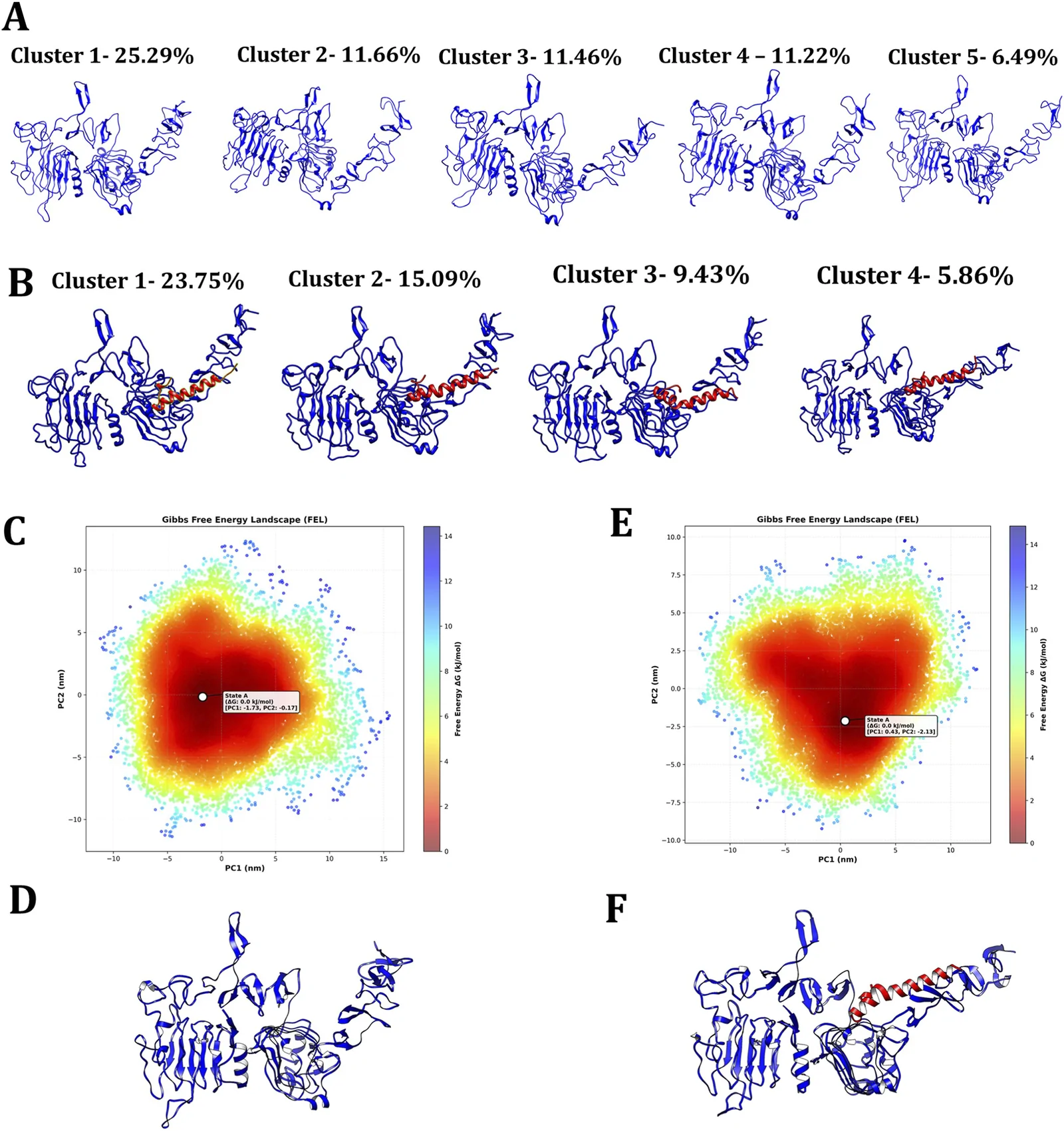
PCA-based conformational clustering and FEL analysis of the 1N8Z receptor and 1N8Z–peptide complex. **(A)** Major PCA-derived conformational clusters of apo 1N8Z. **(B)** Major PCA-derived conformational clusters of the 1N8Z–peptide complex. **(C)** Two-dimensional FEL of apo 1N8Z projected onto PC1 and PC2. **(D)** Representative structure corresponding to the dominant conformational region of apo 1N8Z. **(E)** Two-dimensional FEL of the 1N8Z–peptide complex projected onto PC1 and PC2. **(F)** Representative structure corresponding to the dominant conformational region of the 1N8Z–peptide complex.

**FIGURE 10:**
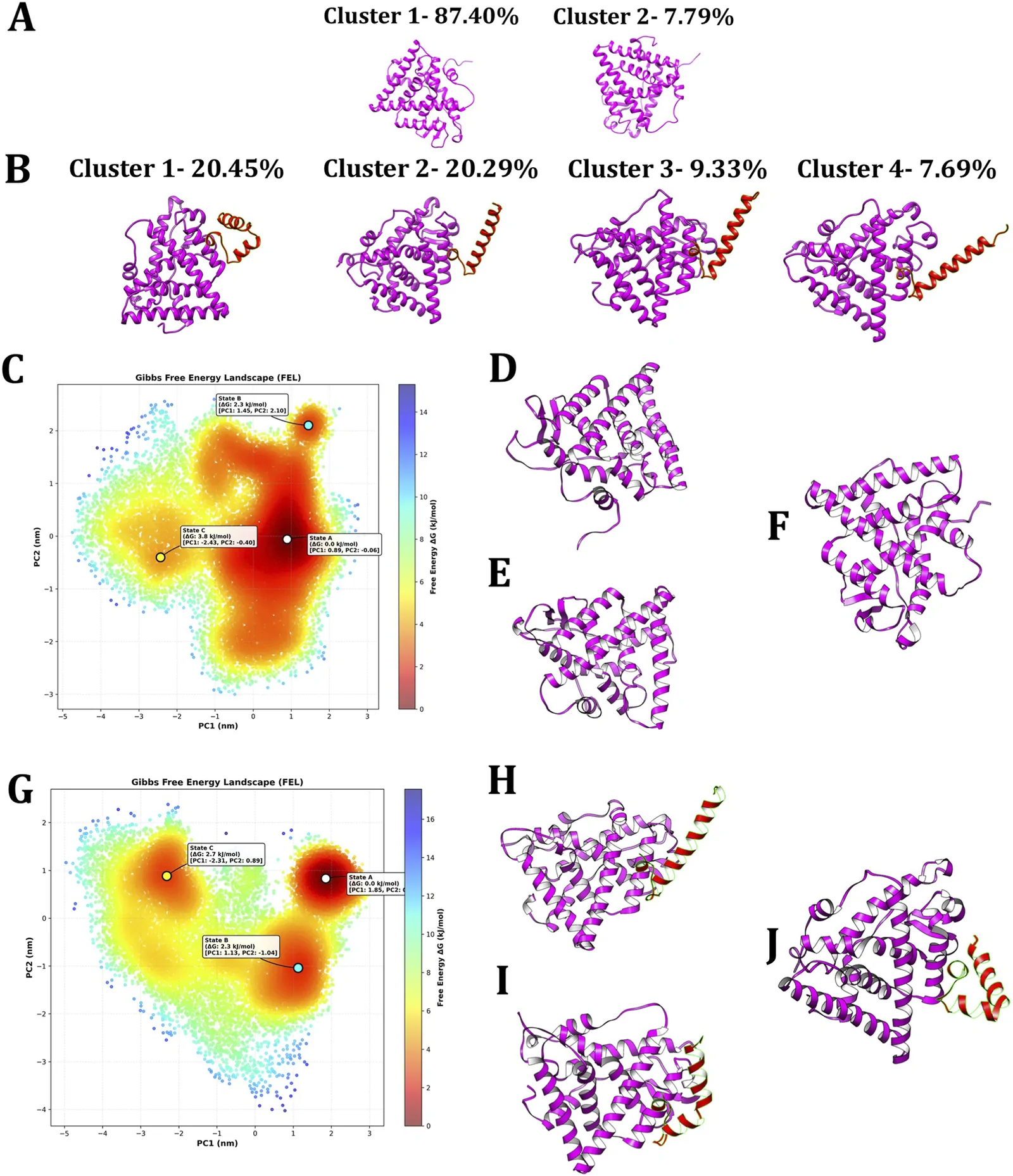
Principal component analysis-based conformational clustering and representative structural sampling of the 3ERT system. **(A)** Principal conformational clusters identified for the apo 3ERT protein, with Cluster 1 and Cluster 2 representing 87.40% and 7.79% of the sampled conformational ensemble, respectively. **(B)** Principal conformational clusters identified for the 3ERT–peptide complex, with Cluster 1, Cluster 2, Cluster 3, and Cluster 4 representing 20.45%, 20.29%, 9.33%, and 7.69% of the sampled conformations, respectively. **(C)** Two-dimensional Gibbs FEL of the apo 3ERT system projected onto PC1 and PC2, showing the distribution of conformational states and corresponding energy minima. **(D–F)** Representative structures obtained from the principal conformational states of the apo 3ERT system. **(G)** Two-dimensional FEL of the 3ERT–peptide complex projected onto PC1 and PC2, showing multiple low-energy conformational basins. **(H–J)** Representative structures corresponding to the principal conformational states of the 3ERT–peptide complex, illustrating the structural diversity sampled during the simulation.

### 3.7 Comparative dynamics of the HER2 (1N8Z) and ERα (3ERT) complexes

A comparative analysis of the HER2 (1N8Z) and ERα (3ERT) peptide-bound complexes demonstrated that both systems remained structurally stable throughout the molecular dynamics simulations, although distinct differences in their dynamic and energetic behaviours were observed. The RMSD, RMSF, Rg, SASA, hydrogen-bond profiles, PCA, and FEL collectively indicated that both complexes maintained stable conformations following peptide binding. The ERα (3ERT) complex exhibited a slightly more favourable molecular docking score and occupied a comparatively narrower conformational space during PCA and FEL analyses, suggesting reduced conformational variability throughout the simulation. In contrast, the HER2 (1N8Z) complex displayed a broader conformational distribution while maintaining overall structural stability. Despite these differences, both receptor–peptide complexes converged to energetically favourable conformational states during the simulation period.

Importantly, MM/PBSA analysis revealed differences in the energetic contributions governing peptide binding. Although docking predicted a marginally stronger initial binding orientation for the ERα complex, the HER2 complex exhibited a more favourable total binding free energy following molecular dynamics equilibration. Energy decomposition further indicated that this stronger predicted binding was primarily associated with favourable van der Waals interactions, while electrostatic interactions also contributed to complex stability. These findings illustrate that molecular docking and MM/PBSA analyses evaluate different aspects of receptor–ligand interactions and therefore provide complementary rather than identical assessments of binding behaviour. The comparative analyses indicate that the *Lacticaseibacillus casei*-derived peptide formed favourable predicted binding interactions with both HER2 and ERα while exhibiting receptor-specific differences in conformational dynamics and binding energetics. These observations provide a comprehensive computational assessment of peptide recognition across two clinically relevant breast cancer targets.

### 3.8 *In silico* ADMET and toxicity prediction

The *in silico* ADMET and toxicity predictions generated using ADMETLab 3.0 are summarised in Table 4, while the complete computational output is provided in Supplementary Material S1. The peptide was computationally predicted to exhibit no hERG channel blockade and a low probability of mutagenicity (Ames test), carcinogenicity, respiratory toxicity, hepatotoxicity, liver injury, or hematotoxicity according to the prediction models. Conversely, the toxicity models predicted potential risks of nephrotoxicity, skin sensitisation, ototoxicity, and genotoxicity.

**TABLE 4:** Comprehensive ADMET, peptide characterisation, and toxicity profile of the *Lacticaseibacillus casei-*derived peptide.

Category	Parameter	Predicted value/result	Interpretation
Physicochemical property	Molecular weight	4,527.19 Da	High molecular weight characteristic of peptide therapeutics
	logP	−18.423	Strongly hydrophilic
	logD7.4	−1.309	Low lipid partitioning
	TPSA	2,020.98 Å^2^	High polarity and hydrogen bonding capability
	Hydrogen bond acceptors	64	Extensive intermolecular interactions
	Hydrogen bond donors	67	Strong aqueous compatibility
Peptide characteristics	Length	37 aa	Medium-sized peptide
	Net charge	−0.009	Near-neutral charge
	pI	7.141	Neutral physiological behaviour
	Instability index	76.778	Potential susceptibility to degradation
	Aliphatic index	84.324	Moderate thermostability
	Boman index	3.602 kcal/mol	High protein-binding potential
	GRAVY	−1.319	Hydrophilic nature
	Hydrophobic ratio	27%	Low hydrophobicity
Absorption	Oral bioavailability	F− (<20%)	Poor oral absorption
	F+ probability	0.011	Low probability of favourable absorption
	Caco-2 permeability	−6.222 log (cm/s)	Poor membrane permeability
	PAMPA permeability	−6.296 log (cm/s)	Limited passive diffusion
	RRCK permeability	−6.203 log (cm/s)	Low epithelial transport
Distribution	Blood-brain barrier permeability	BBB−	Poor CNS penetration
	BBB + probability	0.436	Limited BBB permeability
Stability	Human blood half-life (natural)	41.94 min	Moderate blood stability
	Human blood half-life (modified)	62.13 min	Impredicted stability
	Mouse intestine half-life	0.008 s	Rapid intestinal degradation
Metabolism	CYP1A2 interaction	Low	Minimal metabolism
	CYP2C9 interaction	Low	Reduced interaction potential
	CYP2C19 interaction	Low	Low CYP-mediated effects
	CYP2D6 interaction	Low	Minimal metabolic liability
	CYP3A4 interaction	Low	Reduced drug-drug interactions
Cardiotoxicity	hERG inhibition	Low risk	Favourable cardiac safety
Hepatic toxicity	Hepatotoxicity	Low risk	Minimal liver toxicity
Mutagenicity	Ames toxicity	Negative	Non-mutagenic
Carcinogenicity	Carcinogenicity	Negative	No carcinogenic tendency
Renal toxicity	Nephrotoxicity	Positive	Requires experimental validation
Genotoxicity	Genotoxicity	Positive	Potential toxicity liability
Cell-penetrating potential	Majority of peptide fragments	Non-CPP	Poor membrane translocation
	Fragment TKLPLPDRETRRIIEDNFQKIQDEI	Weak CPP (SVM = 0.22)	Localized membrane interaction potential
Toxicity prediction (ToxinPred)	Representative fragments	Non-toxic	No toxic stretches identified
	SVM scores	Negative	Favourable safety profile
Allergenicity	AllerTOP v2.1 classification	Probable non-allergen	Low allergenic potential
	Closest homolog	CENPK_HUMAN	Human centromere protein K similarity
Overall assessment	Pharmacokinetic profile	Poor oral and membrane permeability	Typical of peptide therapeutics
	Safety profile	Favourable with few toxicity liabilities	Suitable for further experimental investigation
	Potential route of administration	Non-oral formulations	Injectable or targeted delivery systems may be preferable

Abbreviations: TPSA, topological polar surface area; GRAVY, grand average of hydropathicity; BBB, blood–brain barrier; CPP, cell-penetrating peptide; CYP, cytochrome P450; hERG, human ether-à-go-go-related gene; PAMPA, parallel artificial membrane permeability assay; RRCK, rabbit renal cell kidney permeability model.

The Tox21 pathway predictions indicated a low probability of activation of the estrogen receptor (ER), androgen receptor (AR), aromatase, and peroxisome proliferator-activated receptor gamma (PPAR-γ) signalling pathways, whereas moderate probabilities were predicted for the antioxidant response element (ARE) and p53 stress-response pathways. These findings represent computational predictions generated by the employed ADMET platform and provide a preliminary assessment of the pharmacokinetic and toxicity characteristics of the peptide. Detailed prediction values and individual endpoint analyses are presented in Supplementary Material S1.

The toxicological evaluation suggested no hERG blockage, mutagenicity in the Ames test, carcinogenicity, respiratory toxicity, or major hepatotoxicity, which together point to a good safety profile (Supplementary Material S1, attached for detailed analysis results). Furthermore, there was no excessive risk of liver injury or blood toxicity. Then again, the prediction model identified nephrotoxicity, skin sensitisation, ototoxicity, and genotoxicity as risks, thereby warranting additional experimental tests. Further Tox21 pathway analysis indicated little activation of the estrogen receptor, androgen receptor, aromatase, and PPAR-gamma pathways, whereas stress response pathways related to antioxidant response element (ARE) and p53 were moderately probable. Overall, computational ADMET and toxicity studies revealed that peptides from *L. casei* have the physicochemical and pharmacokinetic attributes of peptide drugs and carry a mostly safe profile. While the identified toxicity issues and low oral bioavailability require follow-up, the data support the potential of the peptide for further experimental research and optimisation.

## 4 Discussion

The present study represents a computational continuation of our previous experimental investigation in which a bacteriocin-producing *Lacticaseibacillus casei* VITCM05 strain was isolated from fermented *Cuminum cyminum*, and its peptide was characterised using MALDI-TOF analysis with demonstrated antimicrobial activity and cytotoxicity against the MDA-MB-231 breast cancer cell line (Jannatul Firdous and Mohanasrinivasan, 2022). Building upon those findings, the current work employed an integrated structural bioinformatics workflow to investigate the predicted interaction of the peptide with two clinically important breast cancer receptors, HER2 and ERα. Unlike many computational studies that focus solely on molecular docking, the present investigation integrates peptide structure prediction, molecular docking, molecular dynamics simulations, MM/PBSA binding free-energy analysis, PCA, FEL analysis, and ADMET prediction, thereby providing complementary structural, dynamic, energetic, and pharmacological information for candidate prioritisation. HER2 and ERα were selected because they represent two of the most extensively validated therapeutic targets in breast cancer. HER2 is overexpressed in approximately 15%–20% of invasive breast cancers and promotes constitutive activation of the PI3K/AKT and MAPK signalling pathways, resulting in enhanced tumour proliferation, angiogenesis, invasion, metastasis, and poor clinical prognosis (Harbeck et al., 2019; Loibl et al., 2021). In contrast, ERα is expressed in nearly 70% of breast cancers and functions as the principal regulator of estrogen-dependent transcriptional programmes controlling tumour cell proliferation and survival in hormone receptor-positive disease (Waks and Winer, 2019). Consequently, identifying molecules capable of interacting with these receptors is of considerable interest for peptide-based drug discovery. Nevertheless, it is important to emphasise that the present computational analyses evaluate predicted molecular interactions rather than receptor inhibition or therapeutic efficacy.

Reliable peptide structure prediction is fundamental to computational drug discovery because the accuracy of downstream molecular docking and molecular dynamics simulations depends directly on the quality of the initial structural model. The peptide structure generated using the PEP-FOLD server exhibited acceptable stereochemical quality, with the majority of residues occupying the most favoured regions of the Ramachandran plot and no residues located within disallowed regions. These observations indicate that the predicted model possessed satisfactory stereochemical geometry for subsequent computational analyses and are consistent with previous peptide modelling studies in which Ramachandran statistics serve as an important quality-control criterion before interaction analyses are undertaken (Laskowski et al., 1993; Lamiable et al., 2016). However, the stereochemical quality of the model should not be interpreted as evidence of structural stability or biological activity but rather as confirmation that the predicted conformation was suitable for computational investigation. Molecular docking demonstrated that the *L. casei*-derived peptide formed favourable predicted interactions with both HER2 and ERα, indicating structural compatibility between the peptide and the receptor binding interfaces. Among the evaluated complexes, ERα (PDB ID: 3ERT) exhibited a slightly more favourable docking score than HER2 (PDB ID: 1N8Z), suggesting marginally stronger predicted binding under rigid-body docking conditions. Comparable docking behaviour has been reported for several antimicrobial peptides investigated against cancer-associated receptors, where favourable docking scores were associated with complementary hydrogen bonding, electrostatic interactions, and hydrophobic contacts that facilitate molecular recognition (Khater and Nassar, 2023; Pratik et al., 2025). Nevertheless, molecular docking represents only an initial estimation of binding orientation and should be interpreted cautiously because conventional scoring functions do not account for receptor flexibility, solvent effects, or dynamic conformational rearrangements.

Detailed analysis of the docked complexes revealed that peptide binding was stabilised through cooperative contributions from hydrogen bonds, electrostatic interactions, and hydrophobic contacts. These intermolecular forces collectively determine protein–peptide recognition by enhancing binding specificity and stabilising receptor–ligand complexes. Similar interaction networks have been reported in previous computational investigations of anticancer peptides, where hydrogen-bond formation and hydrophobic complementarity contributed substantially to stable molecular recognition and subsequent dynamic stability during molecular dynamics simulations (Khater and Nassar, 2023; Shanthappa and Melethadathil, 2025). Interestingly, although both receptors exhibited favourable interaction profiles, the binding interfaces differed in their amino acid composition. HER2 primarily interacted through positively charged residues, whereas ERα exhibited a more distributed interaction network involving charged, polar, and hydrophobic amino acids. These receptor-specific interaction patterns likely reflect intrinsic differences in binding-pocket architecture rather than differences in peptide affinity alone and are consistent with previous observations that structurally distinct receptors frequently utilise different combinations of intermolecular interactions for peptide recognition (Pratik et al., 2025). Furthermore, the biological significance of these interactions should be interpreted within the context of receptor function. HER2 signalling drives tumour progression through persistent activation of proliferative and survival pathways, whereas ERα regulates estrogen-responsive gene expression responsible for hormone-dependent tumour growth (Waks and Winer, 2019; Loibl et al., 2021). Therefore, computational identification of peptides capable of interacting with these receptors provides a rational basis for further investigation. However, the present findings should not be interpreted as evidence that the peptide inhibits receptor signalling or suppresses breast cancer progression. Rather, they demonstrate favourable predicted structural interactions that justify continued biochemical and cellular evaluation of this experimentally identified probiotic-derived peptide.

The molecular docking observations were further evaluated through molecular dynamics (MD) simulations, which provide a more realistic representation of protein–peptide behaviour by incorporating atomic motion, solvent effects, and conformational flexibility. While docking predicts an initial binding pose, MD simulations determine whether that interaction remains stable under dynamic physiological conditions. In the present study, both the HER2–peptide and ERα–peptide complexes maintained stable trajectories throughout the simulation period, indicating that peptide binding was preserved following structural equilibration. The RMSD profiles demonstrated rapid convergence to equilibrium with only minor structural deviations thereafter, suggesting that peptide binding did not induce substantial conformational perturbations in either receptor. Similar RMSD behaviour has been reported for stable peptide–protein complexes and is generally considered indicative of equilibrated molecular systems suitable for energetic evaluation rather than direct evidence of biological activity (Hollingsworth and Dror, 2018; Shanthappa and Melethadathil, 2025). The residue-level flexibility observed through RMSF analysis further supported these findings. As expected, the largest fluctuations occurred within solvent-exposed loop regions, whereas residues directly involved in peptide recognition exhibited comparatively lower mobility throughout the simulations. This pattern is consistent with the intrinsic flexibility of loop regions while demonstrating preservation of the receptor-binding interface during peptide association. Likewise, the Rg profiles indicated that both receptors maintained compact conformations following peptide binding, suggesting that ligand association did not compromise their overall structural integrity. Similarly, the relatively stable SASA profiles indicated only modest changes in solvent exposure after complex formation. Collectively, these analyses demonstrate that peptide binding was accommodated without major alterations to receptor architecture, supporting the structural compatibility predicted during molecular docking.

Hydrogen-bond analysis provided additional insight into the stability of the peptide–receptor complexes. Both systems maintained persistent intermolecular hydrogen bonds throughout the simulations, although the number of hydrogen bonds varied dynamically over time. Such fluctuations are characteristic of biologically relevant protein–peptide interactions and reflect continuous formation and disruption of transient intermolecular contacts rather than instability of the complex. Previous molecular dynamics investigations of therapeutic peptides have similarly demonstrated that persistent yet dynamic hydrogen-bond networks contribute significantly to long-term structural stability and receptor recognition (Genheden and Ryde, 2015). Together with the RMSD, RMSF, Rg, and SASA analyses, these observations indicate that the peptide remained stably associated with both receptors throughout the simulation period.

PCA and FEL analysis further expanded the understanding of receptor conformational dynamics following peptide binding. PCA demonstrated that both receptor complexes sampled relatively restricted conformational spaces during the simulations, indicating limited large-scale collective motions. Although the ERα complex occupied a slightly narrower conformational distribution than the HER2 complex, both systems remained clustered within well-defined regions, suggesting stable conformational behaviour following peptide binding. These findings are consistent with previous studies reporting that restricted conformational sampling reflects dynamic equilibrium rather than excessive structural flexibility (David and Jacobs, 2014).

The FEL analysis complemented these observations by characterising the energetic distribution of conformational states sampled during the simulations. Both complexes converged towards distinct low-energy basins, indicating that energetically favourable conformations were achieved after equilibration. The ERα complex displayed a somewhat deeper and more localised energy minimum, whereas the HER2 complex explored a slightly broader conformational landscape while remaining confined within low-energy regions. These differences most likely reflect intrinsic structural characteristics of the receptors rather than substantial differences in peptide stability. Importantly, both complexes occupied energetically favourable conformations throughout the simulations, supporting the conclusion that peptide binding remained dynamically stable under the simulated conditions (Sittel and Stock, 2018).

To obtain a more comprehensive assessment of binding energetics, MM/PBSA calculations were performed using equilibrated molecular dynamics trajectories. Unlike molecular docking, which estimates binding using simplified scoring functions, MM/PBSA incorporates molecular flexibility, solvent effects, and individual energetic contributions to estimate binding free energy more rigorously (Kumari et al., 2014). Interestingly, although molecular docking ranked the ERα complex slightly higher, MM/PBSA predicted a more favourable total binding free energy for the HER2 complex. Rather than representing contradictory findings, these results illustrate the complementary nature of the two computational approaches. Docking evaluates the compatibility of an initial binding pose, whereas MM/PBSA estimates the energetic stability of the equilibrated complex following dynamic relaxation. Differences in receptor ranking between these methods have been widely reported because each evaluates distinct aspects of molecular recognition (Genheden and Ryde, 2015).

Importantly, decomposition of the MM/PBSA energy components demonstrated that the stronger predicted binding of the HER2 complex was primarily driven by favourable van der Waals interactions, while electrostatic interactions also contributed positively to overall complex formation. This observation revises our initial interpretation and provides a more accurate explanation of the energetic basis of peptide recognition. The predominance of van der Waals interactions suggests that efficient intermolecular packing and hydrophobic complementarity constitute the principal energetic determinants stabilising the HER2–peptide complex, whereas electrostatic interactions contribute cooperatively but are not the dominant driving force. Similar energetic profiles have been reported for several peptide–protein complexes in which non-polar interactions account for the largest proportion of the total binding free energy (Kumari et al., 2014; Genheden and Ryde, 2015). Consequently, the combined interpretation of docking, molecular dynamics, PCA, FEL, and MM/PBSA analyses provides complementary evidence supporting favourable predicted interactions of the *L. casei*-derived peptide with both HER2 and ERα while highlighting the importance of integrating multiple computational approaches rather than relying solely on docking scores.

Beyond receptor binding, the pharmacological potential of peptide therapeutics also depends on favourable pharmacokinetic and toxicity characteristics. The ADMET predictions generated in the present study suggested that the peptide possesses physicochemical properties commonly associated with therapeutic peptides, including a relatively high molecular weight and elevated topological polar surface area. These characteristics were predicted to reduce passive membrane permeability and oral bioavailability, findings that are consistent with the known pharmacokinetic limitations of peptide-based therapeutics (Fosgerau and Hoffmann, 2015). Such properties are common among peptide drugs and often necessitate formulation strategies including cyclisation, PEGylation, nanoparticle encapsulation, or targeted delivery systems to improve systemic stability and bioavailability (Ghadiri et al., 2024).

The computational analyses further predicted a low probability of hERG inhibition, hepatotoxicity, mutagenicity, carcinogenicity, and major cytochrome P450 interactions, suggesting a potentially favourable preliminary pharmacological profile. However, these predictions should be interpreted cautiously because they are derived exclusively from computational models and do not represent experimentally validated pharmacokinetic or toxicological behaviour. Conversely, the prediction models also indicated possible risks of nephrotoxicity, genotoxicity, skin sensitisation, and ototoxicity, emphasising that the safety profile of the peptide remains incompletely characterised. Accordingly, the ADMET results should be considered an initial computational screening tool for candidate prioritisation rather than confirmation of clinical safety. Similar recommendations have been made in previous studies evaluating peptide therapeutics, where computational ADMET prediction serves primarily to guide subsequent experimental investigations rather than replace them (Fosgerau and Hoffmann, 2015; Daba and Elkhateeb, 2024).

A major strength of the present investigation is the integration of multiple complementary computational methodologies within a unified structural bioinformatics framework. By combining peptide structure prediction, molecular docking, molecular dynamics simulations, MM/PBSA free-energy calculations, PCA, FEL analysis, and ADMET prediction, the study provides a more comprehensive assessment of peptide–receptor interactions than conventional docking-based investigations. Furthermore, the computational analyses directly extend our previously published experimental characterisation of the *L. casei* peptide, thereby establishing a logical progression from microbial peptide discovery to target-oriented computational evaluation. This integrative strategy strengthens confidence in the computational predictions while providing a rational framework for prioritising candidates for subsequent experimental validation.

Nevertheless, several limitations should be acknowledged. All observations reported in the present study are based exclusively on computational analyses and therefore represent predicted molecular interactions rather than experimentally verified biological activity. Although the integrated computational approaches consistently supported favourable peptide–receptor interactions, they cannot establish receptor inhibition, anticancer efficacy, or therapeutic benefit. Furthermore, only two clinically relevant breast cancer receptors were investigated, whereas breast cancer progression involves complex signalling networks that may influence peptide activity. Future studies should therefore focus on validating these computational predictions through receptor-binding assays, cytotoxicity and apoptosis analyses, mechanistic investigations of downstream signalling pathways, pharmacokinetic studies, and *in vivo* efficacy and toxicity evaluations. Rational peptide optimisation through sequence engineering, cyclisation, or advanced delivery systems may also enhance pharmacokinetic performance while preserving favourable receptor interactions.

## 5 Conclusion

The present study provides a comprehensive computational evaluation of a peptide previously isolated from *Lacticaseibacillus casei* VITCM05 and extends our earlier experimental findings by investigating its predicted interactions with two clinically relevant breast cancer therapeutic targets, HER2 and ERα. Through an integrated structural bioinformatics workflow comprising peptide structure prediction, molecular docking, molecular dynamics simulations, MM/PBSA binding free-energy analysis, principal component analysis, free energy landscape analysis, and *in silico* ADMET prediction, the study provides complementary structural, dynamic, and energetic insights into the molecular behaviour of the peptide. The computational analyses predicted favourable interactions between the peptide and both HER2 and ERα, while molecular dynamics simulations demonstrated that the peptide–receptor complexes remained structurally stable throughout the simulation period. Although molecular docking predicted a slightly more favourable binding orientation for the ERα complex, MM/PBSA analysis indicated stronger overall binding free energy for the HER2 complex following dynamic equilibration, highlighting the complementary nature of these computational approaches. The ADMET predictions provided preliminary information regarding the pharmacokinetic and toxicity characteristics of the peptide; however, these findings represent computational predictions and should not be interpreted as experimentally validated pharmacological properties.

Importantly, this study does not establish the biological efficacy or therapeutic activity of the peptide but instead provides a robust computational framework for prioritising an experimentally identified probiotic-derived peptide for further investigation. Future studies involving receptor-binding assays, cytotoxicity and apoptosis analyses, pharmacokinetic evaluation, and *in vitro* and *in vivo* validation will be essential to determine its therapeutic potential. Overall, the present work demonstrates the value of integrating experimental peptide discovery with advanced structural bioinformatics approaches to facilitate the early-stage identification and rational development of probiotic-derived peptide therapeutics for breast cancer.

## Data Availability

The original contributions presented in the study are included in the article/Supplementary Material; further inquiries can be directed to the corresponding author.
